# 2-Bromo-4-chloro-6-(cyclo­hexyl­imino­meth­yl)phenol

**DOI:** 10.1107/S1600536811045053

**Published:** 2011-11-02

**Authors:** Dong-Yue Wang, Xu-Feng Meng, Jing-Jun Ma

**Affiliations:** a Hebei Key Laboratory of Bioinorganic Chemistry, College of Sciences, Agricultural University of Hebei, Baoding 071001, People’s Republic of China

## Abstract

The title compound, C_13_H_15_BrClNO, was prepared by the condensation of equimolar quanti­ties of 3-bromo-5-chloro­salicyl­aldehyde with cyclo­hexyl­amine in methanol. There is an intra­molecular O—H⋯N hydrogen bond in the mol­ecule. The cyclo­hexyl ring adopts a chair conformation.

## Related literature

For the coordination chemistry of Schiff base compounds, see: Xu *et al.* (2011 ▶); Suleiman Gwaram *et al.* (2011 ▶); Assey *et al.* (2011 ▶). For standard bond lengths, see: Allen *et al.* (1987 ▶). For similar structures, see: Miura *et al.* (2009 ▶); Damous *et al.* (2011 ▶); Şahin *et al.* (2009 ▶); Orona *et al.* (2011 ▶).
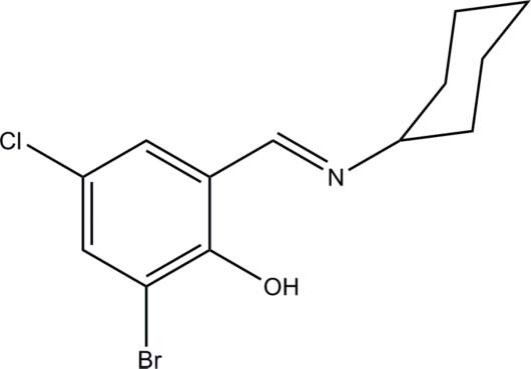


## Experimental

### 

#### Crystal data

C_13_H_15_BrClNO
*M* *_r_* = 316.62
Monoclinic, 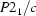

*a* = 12.296 (2) Å
*b* = 16.359 (3) Å
*c* = 6.969 (1) Å
β = 101.634 (2)°
*V* = 1373.0 (4) Å^3^
*Z* = 4
Mo *K*α radiation
μ = 3.17 mm^−1^
*T* = 298 K
0.30 × 0.30 × 0.27 mm

#### Data collection

Bruker SMART 1K CCD area-detector diffractometer
Absorption correction: multi-scan (*SADABS*; Sheldrick, 1996 ▶) *T*_min_ = 0.450, *T*_max_ = 0.481
10912 measured reflections
2982 independent reflections
1705 reflections with *I* > 2σ(*I*)
*R*_int_ = 0.042

#### Refinement

*R*[*F* ^2^ > 2σ(*F* ^2^)] = 0.073
*wR*(*F* ^2^) = 0.227
*S* = 1.04
2982 reflections
157 parameters
1 restraint
H atoms treated by a mixture of independent and constrained refinement
Δρ_max_ = 1.40 e Å^−3^
Δρ_min_ = −0.42 e Å^−3^

### 

Data collection: *SMART* (Bruker, 2007 ▶); cell refinement: *SAINT* (Bruker, 2007 ▶); data reduction: *SAINT*; program(s) used to solve structure: *SHELXS97* (Sheldrick, 2008 ▶); program(s) used to refine structure: *SHELXL97* (Sheldrick, 2008 ▶); molecular graphics: *SHELXTL* (Sheldrick, 2008 ▶); software used to prepare material for publication: *SHELXTL*.

## Supplementary Material

Crystal structure: contains datablock(s) I, global. DOI: 10.1107/S1600536811045053/qm2039sup1.cif

Structure factors: contains datablock(s) I. DOI: 10.1107/S1600536811045053/qm2039Isup2.hkl

Supplementary material file. DOI: 10.1107/S1600536811045053/qm2039Isup3.cml

Additional supplementary materials:  crystallographic information; 3D view; checkCIF report

## Figures and Tables

**Table 1:** Hydrogen-bond geometry (Å, °)

*D*—H⋯*A*	*D*—H	H⋯*A*	*D*⋯*A*	*D*—H⋯*A*
O1—H1⋯N1	0.90 (1)	1.71 (2)	2.564 (6)	159 (6)

**Fig. 1.**
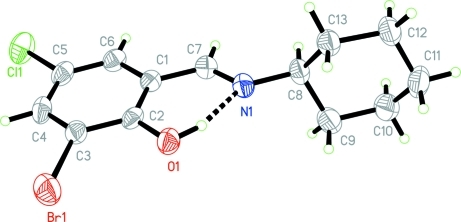
The molecular structure of the title compound, with the numbering scheme and displacement ellipsoids drawn at the 30% probability level. The intramolecular O—H···N hydrogen bond is drawn as a dashed line.

## supplementary crystallographic information

### Comment

Schiff bases are versatile ligands in coordination chemistry (Xu *et al.*, 2011; Suleiman Gwaram *et al.*, 2011; Assey *et al.*, 2011). As a contribution to a structural study on Schiff base compounds, we present here the crystal structure of the title compound, that was obtained as the product of the reaction of 3-bromo-5-chlorosalicylaldehyde with cyclohexylamine in methanol.

In the title compound, Fig. 1, there in an intramolecular O1—H1···N1 hydrogen bond (Table 1). The C1—C6 benzene ring is approximately perpendicular to the C8—C13 cyclohexyl ring. As expected, the cyclohexyl ring adopts a chair conformation. The bond distances and angles are within normal ranges (Allen *et al.*, 1987), and agree well with the corresponding bond distances and angles reported in closely related compounds (Miura *et al.*, 2009; Damous *et al.*, 2011;  Şahin *et al.*, 2009; Orona *et al.*, 2011).

### Experimental

To a methanol solution (10 ml) of 3-bromo-5-chlorosalicylaldehyde (0.1 mmol, 23.5 mg) and cyclohexylamine (0.1 mmol, 9.9 mg), a few drops of acetic acid were added. The mixture was refluxed for 1 h and then cooled to room temperature. The yellow crystalline solid was collected by filtration, washed with cold methanol and dried in air. Single crystals, suitable for X-ray diffraction, were obtained by slow evaporation of a methanol solution of the product in air.

### Refinement

The OH H-atom was located in a difference Fourier map and was refined with a distance restraint, O—H = 0.90 (1) Å, and *U*_iso_(H) = 0.08 Å^2^. The C-bound H atoms were positioned geometrically and refined using a riding model, with C—H = 0.93–0.98 Å, with *U*_iso_(H) = 1.2*U*_eq_(C).

### Crystal data

**Table:**

C_13_H_15_BrClNO	*F*(000) = 640
*M**_r_* = 316.62	*D*_x_ = 1.532 Mg m^−^^3^
Monoclinic, *P*2_1_/*c*	Mo *K*α radiation, λ = 0.71073 Å
*a* = 12.296 (2) Å	Cell parameters from 2374 reflections
*b* = 16.359 (3) Å	θ = 2.5–24.1°
*c* = 6.969 (1) Å	µ = 3.17 mm^−^^1^
β = 101.634 (2)°	*T* = 298 K
*V* = 1373.0 (4) Å^3^	Block, yellow
*Z* = 4	0.30 × 0.30 × 0.27 mm

### Data collection

**Table:**

Bruker SMART 1K CCD area-detector diffractometer	2982 independent reflections
Radiation source: fine-focus sealed tube	1705 reflections with *I* > 2σ(*I*)
graphite	*R*_int_ = 0.042
ω scan	θ_max_ = 27.0°, θ_min_ = 2.5°
Absorption correction: multi-scan (*SADABS*; Sheldrick, 1996)	*h* = −15→15
*T*_min_ = 0.450, *T*_max_ = 0.481	*k* = −20→19
10912 measured reflections	*l* = −8→8

### Refinement

**Table:**

Refinement on *F*^2^	Primary atom site location: structure-invariant direct methods
Least-squares matrix: full	Secondary atom site location: difference Fourier map
*R*[*F*^2^ > 2σ(*F*^2^)] = 0.073	Hydrogen site location: inferred from neighbouring sites
*wR*(*F*^2^) = 0.227	H atoms treated by a mixture of independent and constrained refinement
*S* = 1.04	*w* = 1/[σ^2^(*F*_o_^2^) + (0.1329*P*)^2^ + 0.243*P*] where *P* = (*F*_o_^2^ + 2*F*_c_^2^)/3
2982 reflections	(Δ/σ)_max_ < 0.001
157 parameters	Δρ_max_ = 1.40 e Å^−^^3^
1 restraint	Δρ_min_ = −0.42 e Å^−^^3^

### Special details

**Table:**

Geometry. All e.s.d.'s (except the e.s.d. in the dihedral angle between two l.s. planes) are estimated using the full covariance matrix. The cell e.s.d.'s are taken into account individually in the estimation of e.s.d.'s in distances, angles and torsion angles; correlations between e.s.d.'s in cell parameters are only used when they are defined by crystal symmetry. An approximate (isotropic) treatment of cell e.s.d.'s is used for estimating e.s.d.'s involving l.s. planes.
Refinement. Refinement of *F*^2^ against ALL reflections. The weighted *R*-factor *wR* and goodness of fit *S* are based on *F*^2^, conventional *R*-factors *R* are based on *F*, with *F* set to zero for negative *F*^2^. The threshold expression of *F*^2^ > σ(*F*^2^) is used only for calculating *R*-factors(gt) *etc*. and is not relevant to the choice of reflections for refinement. *R*-factors based on *F*^2^ are statistically about twice as large as those based on *F*, and *R*- factors based on ALL data will be even larger.

### Fractional atomic coordinates and isotropic or equivalent isotropic displacement parameters (Å^2^)

**Table:**

	*x*	*y*	*z*	*U*_iso_*/*U*_eq_	
Br1	0.36373 (6)	0.05122 (4)	−0.11130 (11)	0.0888 (4)	
Cl1	0.09344 (12)	0.31936 (13)	−0.2918 (3)	0.0948 (6)	
N1	0.6092 (4)	0.3288 (3)	0.0492 (7)	0.0594 (11)	
O1	0.5335 (3)	0.1823 (2)	0.0130 (6)	0.0619 (9)	
C1	0.4189 (4)	0.3005 (3)	−0.0749 (6)	0.0479 (11)	
C2	0.4337 (4)	0.2154 (3)	−0.0606 (6)	0.0490 (11)	
C3	0.3424 (4)	0.1648 (3)	−0.1214 (7)	0.0550 (12)	
C4	0.2398 (4)	0.1959 (4)	−0.1955 (7)	0.0624 (14)	
H4	0.1801	0.1612	−0.2402	0.075*	
C5	0.2255 (4)	0.2799 (4)	−0.2032 (7)	0.0616 (14)	
C6	0.3129 (4)	0.3316 (3)	−0.1472 (7)	0.0576 (13)	
H6	0.3020	0.3878	−0.1572	0.069*	
C7	0.5119 (4)	0.3548 (3)	−0.0185 (7)	0.0564 (12)	
H7	0.5003	0.4109	−0.0322	0.068*	
C8	0.7014 (4)	0.3862 (3)	0.0990 (8)	0.0601 (13)	
H8	0.6719	0.4419	0.0785	0.072*	
C9	0.7837 (5)	0.3731 (4)	−0.0324 (9)	0.0809 (18)	
H9A	0.7478	0.3837	−0.1672	0.097*	
H9B	0.8083	0.3167	−0.0231	0.097*	
C10	0.8835 (6)	0.4293 (5)	0.0252 (12)	0.095 (2)	
H10A	0.9367	0.4179	−0.0569	0.114*	
H10B	0.8598	0.4857	0.0036	0.114*	
C11	0.9385 (5)	0.4176 (4)	0.2383 (11)	0.089 (2)	
H11A	0.9677	0.3625	0.2579	0.107*	
H11B	1.0000	0.4555	0.2730	0.107*	
C12	0.8578 (5)	0.4318 (5)	0.3658 (9)	0.0772 (18)	
H12A	0.8343	0.4885	0.3550	0.093*	
H12B	0.8937	0.4218	0.5010	0.093*	
C13	0.7562 (5)	0.3768 (4)	0.3122 (8)	0.0674 (14)	
H13A	0.7782	0.3203	0.3381	0.081*	
H13B	0.7033	0.3907	0.3934	0.081*	
H1	0.576 (4)	0.227 (2)	0.035 (9)	0.080*	

### Atomic displacement parameters (Å^2^)

**Table:**

	*U*^11^	*U*^22^	*U*^33^	*U*^12^	*U*^13^	*U*^23^
Br1	0.0837 (6)	0.0650 (5)	0.1181 (7)	−0.0128 (3)	0.0212 (4)	−0.0034 (3)
Cl1	0.0463 (8)	0.1216 (15)	0.1084 (13)	0.0132 (8)	−0.0034 (8)	0.0139 (11)
N1	0.050 (2)	0.062 (3)	0.063 (3)	−0.004 (2)	0.0047 (19)	−0.006 (2)
O1	0.0451 (19)	0.063 (2)	0.076 (2)	0.0007 (16)	0.0071 (17)	−0.0017 (18)
C1	0.045 (2)	0.059 (3)	0.039 (2)	0.003 (2)	0.0040 (19)	0.003 (2)
C2	0.039 (2)	0.071 (3)	0.038 (2)	0.004 (2)	0.0115 (18)	0.002 (2)
C3	0.054 (3)	0.064 (3)	0.050 (3)	−0.009 (2)	0.016 (2)	−0.004 (2)
C4	0.042 (3)	0.089 (4)	0.054 (3)	−0.015 (3)	0.005 (2)	0.002 (3)
C5	0.042 (3)	0.091 (4)	0.052 (3)	0.007 (3)	0.007 (2)	0.006 (3)
C6	0.051 (3)	0.066 (3)	0.054 (3)	0.011 (3)	0.009 (2)	0.006 (2)
C7	0.055 (3)	0.056 (3)	0.057 (3)	0.000 (2)	0.010 (2)	0.002 (2)
C8	0.050 (3)	0.048 (3)	0.076 (4)	−0.006 (2)	−0.001 (2)	−0.001 (2)
C9	0.082 (4)	0.088 (4)	0.075 (4)	−0.025 (3)	0.022 (3)	−0.019 (3)
C10	0.082 (5)	0.108 (5)	0.104 (6)	−0.033 (4)	0.039 (4)	−0.026 (4)
C11	0.048 (3)	0.088 (4)	0.127 (6)	−0.008 (3)	0.009 (4)	−0.006 (4)
C12	0.064 (4)	0.086 (4)	0.077 (4)	−0.020 (3)	0.003 (3)	−0.011 (3)
C13	0.062 (3)	0.073 (4)	0.067 (3)	−0.016 (3)	0.015 (3)	−0.008 (3)

### Geometric parameters (Å, °)

**Table:**

Br1—C3	1.875 (5)	C8—C13	1.510 (7)
Cl1—C5	1.741 (5)	C8—H8	0.9800
N1—C7	1.268 (6)	C9—C10	1.521 (8)
N1—C8	1.460 (6)	C9—H9A	0.9700
O1—C2	1.344 (5)	C9—H9B	0.9700
O1—H1	0.900 (10)	C10—C11	1.515 (10)
C1—C6	1.395 (7)	C10—H10A	0.9700
C1—C2	1.405 (7)	C10—H10B	0.9700
C1—C7	1.440 (7)	C11—C12	1.478 (9)
C2—C3	1.391 (7)	C11—H11A	0.9700
C3—C4	1.363 (7)	C11—H11B	0.9700
C4—C5	1.384 (8)	C12—C13	1.524 (7)
C4—H4	0.9300	C12—H12A	0.9700
C5—C6	1.362 (7)	C12—H12B	0.9700
C6—H6	0.9300	C13—H13A	0.9700
C7—H7	0.9300	C13—H13B	0.9700
C8—C9	1.510 (8)		
			
C7—N1—C8	120.1 (5)	C8—C9—H9A	109.4
C2—O1—H1	101 (4)	C10—C9—H9A	109.4
C6—C1—C2	119.1 (4)	C8—C9—H9B	109.4
C6—C1—C7	120.4 (5)	C10—C9—H9B	109.4
C2—C1—C7	120.5 (4)	H9A—C9—H9B	108.0
O1—C2—C3	119.7 (5)	C11—C10—C9	111.0 (6)
O1—C2—C1	121.4 (4)	C11—C10—H10A	109.4
C3—C2—C1	118.9 (4)	C9—C10—H10A	109.4
C4—C3—C2	121.5 (5)	C11—C10—H10B	109.4
C4—C3—Br1	119.7 (4)	C9—C10—H10B	109.4
C2—C3—Br1	118.7 (4)	H10A—C10—H10B	108.0
C3—C4—C5	119.1 (5)	C12—C11—C10	110.4 (5)
C3—C4—H4	120.5	C12—C11—H11A	109.6
C5—C4—H4	120.5	C10—C11—H11A	109.6
C6—C5—C4	121.3 (4)	C12—C11—H11B	109.6
C6—C5—Cl1	119.8 (5)	C10—C11—H11B	109.6
C4—C5—Cl1	118.9 (4)	H11A—C11—H11B	108.1
C5—C6—C1	120.1 (5)	C11—C12—C13	112.2 (5)
C5—C6—H6	119.9	C11—C12—H12A	109.2
C1—C6—H6	119.9	C13—C12—H12A	109.2
N1—C7—C1	122.2 (5)	C11—C12—H12B	109.2
N1—C7—H7	118.9	C13—C12—H12B	109.2
C1—C7—H7	118.9	H12A—C12—H12B	107.9
N1—C8—C9	110.4 (4)	C8—C13—C12	111.2 (5)
N1—C8—C13	109.8 (4)	C8—C13—H13A	109.4
C9—C8—C13	111.2 (5)	C12—C13—H13A	109.4
N1—C8—H8	108.4	C8—C13—H13B	109.4
C9—C8—H8	108.4	C12—C13—H13B	109.4
C13—C8—H8	108.4	H13A—C13—H13B	108.0
C8—C9—C10	111.0 (5)		

### Hydrogen-bond geometry (Å, °)

**Table:**

*D*—H···*A*	*D*—H	H···*A*	*D*···*A*	*D*—H···*A*
O1—H1···N1	0.90 (1)	1.71 (2)	2.564 (6)	159 (6)
